# The Use of “Retardation” in FRAXA, FMRP, FMR1 and Other Designations

**DOI:** 10.3390/cells11061044

**Published:** 2022-03-19

**Authors:** Jonathan Herring, Kirsten Johnson, Jörg Richstein

**Affiliations:** 1 Faculty of Law, Oxford University, Oxford OX1 3UL, UK; jonathan.herring@law.ox.ac.uk; 2 The Fragile X Society, Great Dunmow CM6 1DA, UK; 3 Fragile X International, Egmont St 11, B-1000 Brussels, Belgium; jr@frax.de; 4 Interessengemeinschaft Fragile-X e.V, 18055 Rostock, Germany

**Keywords:** fragile X syndrome (FXS), fragile X premutation associated conditions (FXPAC), FRAXA, FMRP, FMR1, FMR2, FXR1, FXR2

## Abstract

The European Fragile X Network met in Wroclaw, Poland, November 2021, and agreed to work towards the eradication of the word “retardation” in regard to the naming of the fragile X gene (FRAXA) and protein (FMRP). There are further genes which have “retardation” or abbreviations for “retardation” in their names or full designations, including FMR1, FMR2, FXR1, FXR2, NUFIP1, AFF1, CYFIP1, etc. “Retardation” was commonly used as a term in years past, but now any reference, even in an abbreviation, is offensive. This article discusses the stigmatisation associated with “retardation”, which leads to discrimination; the inaccuracy of using “retardation” in these designations; and the breadth of fragile X syndrome being beyond that of neurodiversity. A more inclusive terminology is called for, one which ceases to use any reference to “retardation”. Precedents for offensive gene names being altered is set out. The proposal is to approach the HGNC (HUGO [Human Genome Organisation] Gene Nomenclature Committee) for new terminology to be enacted. Ideas from other researchers in the field are welcomed.

## 1. Setting Out the Issue

The moment of diagnosis has come. The patient waits to hear their fate. Generally, the least of a patient’s concern is the exact medical terminology used to define a condition’s characteristics. Of far more significance will be the prognosis and treatment options. Yet, the name attached to a condition and indeed whether it is given a name at all can carry huge significance. The person will inevitably inform their friends, employers and family of the condition with the medical name. It will become a staple of their internet search history. The terminology used can convey negative (or positive) connotations. It may provide a person with reassurance that they can now put a label onto the array of symptoms they have identified and indicate fellowship with others with the same condition or gene. However, the label may also carry connotations which are inaccurate or convey negative stereotypes. It is this latter issue with which we are primarily concerned. There is historical precedence for changing the name of genes which have been named inaccurately, due to the first associations or functions discovered for that gene. Examples will be given later in this article.

The European Fragile X Network, which consists of seventeen different national fragile X associations, met in Wroclaw, Poland, November 2021, and agreed to work towards the eradication of the word “retardation” in regard to the naming of the fragile X gene and protein.

The full registered name of the fragile X gene is FRAXA (Fragile X Site, Folic Acid Type, Rare, Fra(X) (Q27.3) A (Macroorchidism, Mental Retardation)) [1,2], though it is more commonly referred to as FMR1. The protein which codes for this gene is called FMRP (fragile X mental retardation protein) [3].

A complete list of terms using “retardation” or abbreviations for “retardation” in their names or full designations are:

- FRAXA
- FMRP
- FMR1 (FMRP translational regulator 1) [4,5]
- FMR2 (familial mental retardation protein 2) [6]
- FMR1-AS1 (FMR1 antisense RNA 1) [7]
- FMR1NB (FMR1 neighbor) [8]
- FXR1 (FMR1 autosomal homolog 1) [9]
- FXR2 (FMR1 autosomal homolog 2) [10]
- NUFIP1 (nuclear FMR1 interacting protein 1) [11]
- NUFIP2 (nuclear FMR1 interacting protein 2) [12]
- AFF1 (AF4/FMR2 family member 1) [13]
- AFF2 (AF4/FMR2 family member 2) [14]
- AFF3 (AF4/FMR2 family member 3) [15]
- AFF4 (AF4/FMR2 family member 4) [16]
- CYFIP1 (cytoplasmic FMR1 interacting protein 1) [17]
- CYFIP2 (cytoplasmic FMR1 interacting protein 2) [18]
- FMR1-IT1 (FMR1 intronic transcript 1) [19]

“Retardation” was commonly used as a term in years past throughout the scientific and clinical literature by convention, and perhaps without careful consideration of the term. Now, however, any reference, even in an abbreviation, is offensive.

There are three dangers that can arise with this labelling.

**i. Stigmatisation**

The use of the word “retardation” is highly stigmatic. Indeed, even back in 2007 in a survey by the British Broadcasting Corporation (BBC) of what were the worst words to describe those with disabilities, “retard” was the top one [20]. A study of American doctors found that “the majority of parents indicated that they would be upset if a physician used the term mental retardation. Some professionals reported being criticized for using the term” [21]. An international campaign has been operating for many years now to stop the use of the words “retard” and “retarded” [22], a campaign supported by the Special Olympics. The issue has been addressed in the media: in 2011 and 2013, articles in the Washington Post [23] and New York Times [24] discouraged the use of the term. Indeed, in the United States, “Rosa’s Law” means that as a matter of federal law “mental retardation” should be referred to as “intellectual disability” for all legal purposes [25]. In 2013, The Diagnostic and Statistical Manual of Mental Disorders, Fifth Edition (DSM-5) changed the terminology “mental retardation” to “intellectual disability” [26]. The updated International Classification of Diseases (ICD-11) was adopted in 2019 and implemented on 1 January 2022 [27]. Unfortunately, “retardation” is still extensively used in the gene and protein nomenclature connected to fragile X [28,29].

Doctors explaining fragile X syndrome (FXS) and fragile X premutation associated conditions (FXPAC) [30] to patients will be referencing terms which contain the word, retardation, that in any other context would be found to express contempt and provoke outrage. Children being told of their condition by doctors will find it carries a word they will only have heard in the context of bullying in the playground. This is not just an English-language problem: for example, in German, the word “retardiert” translates to “zurückgeblieben”, which is again very offensive and, also, simply wrong.

Fragile X is not alone in this respect—other examples can be found of conditions or genes with the word retardation in them, such as hyperphosphatasia with mental retardation syndrome (HPMRS) [31], and Claes-Jensen-type syndromic mental retardation [32].

Further, Link and Phelan added the concept of discrimination to stigma. They stated stigma is present “when elements of labelling, stereotyping, separation, status loss, and discrimination co-occur in a power situation” [33] (p. 367). In regard to fragile X, it is frankly astonishing that such offensive and stigmatising terminology is still thought appropriate and used without apology or explanation. Of course, it is fair to note that there is much more to challenging stigma than “changing words” [34]. The notion that the alteration of a label will remove prejudice against people with a particular condition is clearly fanciful and undermines the severity of the issues raised. Nevertheless, it is clear that certain forms of terminology will inflame disadvantage and draw on negative connotations, as we have seen recently with racially offensive epithets. New names can shine a light on a positive aspect of a condition or educate. An example might be the widespread acceptance of the autism spectrum which informs that autism appears in different forms and should not be understood as a static entity of characteristics.

**ii. Misleading: Inaccurate**

FRAXA itself is a perfectly healthy gene to have, and FMRP is a necessary protein. It is only the gene’s alteration (with extended repeats leading to either too much mRNA or the protein not being produced) that results in FXPAC and FXS. It is simply inaccurate to define the gene in terms of two things (macroorchidism and retardation) which happen when it goes wrong. FMRP is an important protein which is present in everyone. It seems particularly significant in the operation of the brain, testes and ovaries. While the full understanding of the protein is still being developed, it is already known to assist the brain in making connections between cells through synapses, where communication between cells take place. In particular, FMRP helps regulate synaptic plasticity [35]. However, this protein is defined in terms of how it operates in rare cases, when there are high numbers of repeats. This is similar to describing the brain as the dementia organ [36].

**iii. Misleading: Over-Focused**

Referring to fragile X syndrome as “retardation” is not only offensive but is also profoundly misleading. First, while many of those with FXS will have learning difficulties, sometimes severe, plenty of children, particularly girls with FXS, will not have that symptom. Physical manifestations in those with FXS may include hypotonia, mitral valve prolapse, seizures, gastro reflux, hernias, urinary reflux, constipation, etc. [37]. There are behavioural issues such as ADHD, and psychological difficulties such as anxiety [38]. Fragile X syndrome is far more complex than intellectual disability. “Retardation” is narrow, focusing on a single dimension that affects some people with FXS and ignores their many other characteristics, including both difficulties and strengths.

Other features of FXS include short attention span, distractibility, impulsiveness, restlessness, over activity, sensory, emotional and communication difficulties. FXS often includes features such as excellent long-term memory, good imitation skills, likeable personalities, being sensitive to others and having a strong sense of humour [38]. There are a range of physical characteristics, including a long narrow face and prominent jaw bones and ears [39]. Many of those with FXS show features of the autism spectrum [40]. By describing FXS in terms of a learning disability, the condition is misleadingly reduced to a single characteristic.

After thirty years of research, FXS is now understood in far more detail, and its effect on the body is known to be broader than that of intellectual disability. By using “retardation”, the emphasis is on the learning disability aspect of fragile X, rather than seeing FXS as a chromosomal condition which has a range of effects across girls/women and boys/men.

## 2. Towards a More Inclusive Terminology

We have established there are problems with current nomenclature. Retardation is now seen as an offensive word and has been removed from the Center for Disease Control descriptions of any disease, condition or syndrome relating to intellectual disability [41]. Intellectual disability or learning disability is preferred nowadays, with perhaps neurodiversity being a better umbrella term. Continuing to use the word “retardation” in the naming of this gene and protein is derogatory, stigmatising, and morally wrong. It is not an inclusive term. It limits the understanding of FXS to intellectual disability when the way this gene plays out in individuals can vary widely. In addition, those with fragile X premutation associated conditions, which are caused by the premutation of this FRAXA gene, have reported that they feel offended by the link to “retardation” being part of the gene’s name as these conditions have no link to intellectual disability.

It should be noted that current guidelines on the naming of genes say that “nomenclature should not be offensive or pejorative” [42]. The European Fragile X Network organisations therefore propose that FRAXA, and associated terms, should be renamed. Any reference to retardation should be removed. Additionally, macroorchidism should undoubtedly be removed from the current FRAXA definition as it does not apply to females with FXS. We suggest that:

- FRAXA is modified to Fragile X Site, Folic Acid Type, Rare, Fra(X) (Q27.3) A
- FMRP becomes FXP (fragile X protein)
- FMR1 becomes FX1 or FXTR1 (fragile X translational regulator 1)
- FMR2 becomes FP2 (familial protein 2)

Many of the fragile X terms listed at the start of this article have had “retardation” removed from their full definition, with only FMR1 or FMR2 included in the official definitions. However, in line with changing FMR1 and FMR2 to more inclusive terminology, the updated versions of FMR1 and FMR2 should be used in other gene definitions.

## 3. How Do We Make This Happen?

It is proposed that the authors and Fragile X International [43], with the support of the scientific and research community, will approach the HGNC (HUGO [Human Genome Organisation] Gene Nomenclature Committee), requesting a change of all of the terms related to fragile X which include the word “retardation” or references to terms which include “MR” (mental retardation). There is a process in place and precedent for these changes being made. If anyone would like to add their name to this application for name change, or support our efforts with advice and ideas, please let us know.

A very good example of a change which has already happened is with NUFIP1P1 (NUFIP1 pseudogene 1) [44], which was previously defined as “nuclear fragile X mental retardation protein interacting protein 1 pseudogene”, but all references to retardation have been removed.

Another example of a gene name which has been altered is Pettigrew syndrome (PGS). This was previously Pettigrew X-linked mental retardation syndrome, but is now referred to as PGS, or more fully as X-linked Dandy-Walker malformation with intellectual disability, basal ganglia disease and seizures (XDIBS) [45], with the gene referenced as AP1S2 [46]. Further, the “Mental retardation, X-linked 72” gene (Waisman syndrome [47], which has been described also as basal ganglion disorder with mental retardation), is now termed RAB39B [48]. There is a group of genes with a protein product previously labelled as “mental retardation X-linked” (MRXS1; MRX76; MRX54; MRX43; MRX36; MRX29; MRX32; MRX33; MRX38; MRX87) now placed under the ARX (aristaless-related homeobox) term [49]. Moreover, there is the non-offensive HDAC4, updated from the previously known brachydactyly mental retardation syndrome [50]. The gene for Sutherland-Haan X-linked mental retardation syndrome, today commonly referred to as Sutherland-Haan syndrome, is now PQBP1 [51]. It is yet another of the X-linked mental retardation (XLMR) syndromes, all of which should have their terminology updated if that has not yet been done.

## 4. Conclusions

If a parent were to tell their non-medically qualified friend that their child has “fragile X mental retardation protein” deficiencies, a message is being sent in the words used. Preconceptions are being formed without that child being accepted for who they are by the friend, and without the friend having an unbiased acceptance of the condition fragile X syndrome as it affects that child. The stigma and discrimination begin at birth. Without the attachment of the term “retardation”, FXS and FXPAC can be seen as chromosomal conditions in and of themselves, affecting different people in different ways, and indeed the same person across their lifespan in different ways.

Why should we limit those with FXS and FXPAC? We have scientific studies and much anecdotal evidence from families that acceptance and enablement do far more good than imposing pre-conceived limitations and a top-down treatment approach to “fixing” a problem. Inclusion and coming alongside are far more helpful. Changing this terminology will make the world of difference to how those with FXS and FXPAC are viewed, treated, and accepted in wider society.
